# Bullying, shortage of staff and resources in workplace: Qualitative experience of newly qualified nurses

**DOI:** 10.4102/curationis.v46i1.2407

**Published:** 2023-03-31

**Authors:** Agrinette N. Madolo, Siyathemba P. Hloba

**Affiliations:** 1 Department of Nursing, Faculty of Health Sciences, Walter Sisulu University, Mthatha, South Africa

**Keywords:** bullying, novice professional, emotional impact, overwhelmed, minimal experience

## Abstract

**Background:**

The results of the study conducted at Alfred Nzo Municipality revealed that newly qualified nurses were overwhelmed with the challenges surrounding the execution of their duties in healthcare facilities. The experienced staff largely ignored the newly appointed personnel, which led to emotional distress among the newly qualified nurses.

**Objectives:**

This study aimed to explore and describe the effects of bullying and the shortage of staff and resources in the workplace on newly qualified nurses and to evaluate the support offered to these nurses in the workplace.

**Method:**

A qualitative, explorative, descriptive and contextual design was used with semi-structured interviews to gather data that were analysed using Tesch’s thematic analysis.

**Results:**

The themes that emerged revealed that the participants felt bullied in the workplace, the shortage of staff and resources made the participants feel ineffective in their work environment, and the clinical exposure to different units and procedures added value to the participants’ development in the workplace.

**Conclusion:**

The study revealed that bullying has adverse implications for newly qualified staff. The shortage of staff and resources made the newly qualified nurses feel ineffective and useless but their rotation through the wards added value to their development and confidence in their expertise.

**Contribution:**

A conceptual framework serves as a guide to newly qualified professional nurses in guiding, protection and coaching in the workplace.

## Introduction

The Eastern Cape Provincial Department of Health provides financial support to all institutions that train nurses in the province to ensure that an adequate number of nursing personnel are produced to ensure quality patient care. The newly qualified nursing professionals produced by these institutions are handed over to the clinical services for employment as novice professional nurses but these people often encounter bullying and a shortage of staff and resources in the workplace. The Department of Health, like any other organisation, has to maintain a certain standard by which it can be measured in its effort to ensure quality nursing care. This means that while the department is trying, by all means possible, to develop and groom newly qualified nurses, it must simultaneously provide a high level of healthcare for society. While the department is attempting to balance these two objectives, newly qualified nurses may experience an opportunity to reinforce implementation plans or fill gaps that need a quality improvement plan. A conceptual framework was developed to guide the discussion of bullying and the shortage of staff and resources in the workplace.

## Problem statement

Based on the researcher’s experience while working as a professional nurse, a large percentage of newly qualified professional nurses resign from the nursing service after completing their community service to pursue various other career paths. This triggered the researcher’s interest in exploring the clinical experiences of newly qualified professional nurses in hospital facilities and found that limited means were available to follow up on the nurses’ clinical experiences in the workplace that could motivate or demotivate them in their daily work.

### Aim

This study aimed to explore and describe the bullying behaviour of senior nurses and the shortage of staff and resources in the workplace.

### The rationale of the study

This study was anticipated to assist in guiding newly qualified nursing professionals to gain support from the Department of Health. The nursing education institutions could learn from the senior staff members how to deal with bullying and the shortage of staff and resources and identify gaps in their curricula. The multidisciplinary team may gain insight into the newly qualified professional nurses’ clinical experiences to strengthen their assistance in the work environment.

This study attempted to assist hospital management by highlighting the gaps in the support system and strengthening strategies to enhance the newly qualified professional nurses’ well-being. The study was anticipated to serve as a reference for future research studies and provide evidence-based practice in nursing.

### Conceptual framework

A conceptual framework represents the researcher’s synthesis of the literature to explain a phenomenon (Regoniel 2015:1). It also maps out the actions required in the course of the study, the researcher’s previous knowledge and observations, and other researchers’ points of view.

Fawcett and DeSanto-Madeya’s (2013:6) theoretical model was used to provide a frame of reference for the bullying encountered by newly qualified professional nurses in hospital facilities. The theoretical model describes how human beings cope with their health needs and the environment surrounding them in healthcare facilities concerning the nursing care they receive. The model maintains that human beings, both healthy and suffering, are the main focus of care provision to enhance their well-being and proceeds to argue that human beings want to be treated in a safe and peaceful environment. The authors state that human beings need to be taught and educated about their health, for example, the warning signs of ill health and lifestyle consequences. Finally, the model argues that nursing practice is the main factor in caring for those in need.

The combination of art and science in the nursing discipline could assist in achieving the nursing goal of caring for both the healthy and the suffering (Fawcett & DeSanto-Madeya 2013:6). That is only achieved when following the regulations that guide nursing as a profession. These observations and arguments led the researchers to identify four concepts, namely human beings, environment, health and nursing as the foundation for their conceptual model for advanced clinical practice.

## Research methods and design

### Description of concepts

**FIGURE 1:**
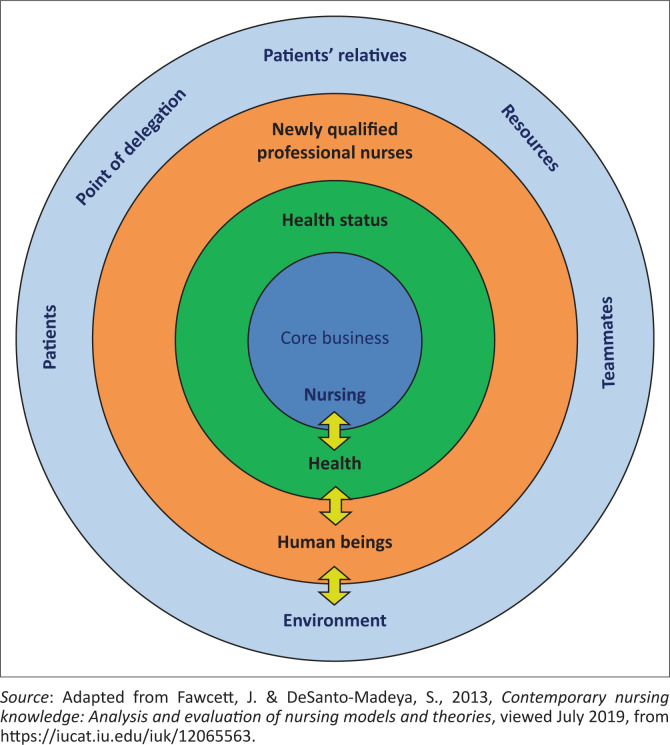
A conceptual model.

The concepts that were utilised as a structure to explain the newly qualified professional nurses’ experiences in hospital facilities are described as follows (Figure 1):

**Environment** (external conditions or surroundings): As newly qualified professional nurses are human beings, they also need to live in an environment conducive to their well-being. This concept refers to the four hospital facilities that were chosen for this study and applied to the participants’ relationships with teammates, patients and their relatives in the clinical units in which they were tasked to carry out their duties. This concept includes the resources available and the points of delegation for the participants to execute their duties.

The subconcepts described hereunder were used to measure the environment to which the newly qualified professional nurses were exposed to in the daily execution of their tasks.

**Teammates**: This concept refers to the professionals who interacted with newly qualified nurses daily to provide quality healthcare. These professionals might have been supervisors, colleagues, subordinates or members of multidisciplinary teams.

**Resources:** It refers to the stock or supply of budget, materials, staff and other assets that a hospital facility may need to function effectively (Cambridge Dictionary 2020).

**Patients’ relatives** (those people related to a person receiving medical care): According to Anadolu Medical Center (2015:1), patients’ relatives means the preferred consanguineal and conjugate next of kin to the healthcare service user. This subconcept includes parents, grandparents, siblings, children, marriage partners or friends.

**Point of delegation:** The area in which one is assigned a specific task or purpose (Juneja 2015:1).

**Patients** (a person receiving medical care): This subconcept includes all the people who use hospital services as patients, healthy persons, families, relatives, communities and populations in general, not focusing solely on patients (World Health Organization 2019:1).

**Newly qualified professional nurse**: A beginner professional nurse practitioner and midwife who has the necessary knowledge, skills, attitudes and values to provide an efficient and professional healthcare service (Morolong & Chabeli 2021:1). In this study, the term newly qualified professional nurse was used when referring to a registered nurse with 2 years of experience, excluding the community service period.

**Health status**: An individual’s relative level of wellness and illness, considering the presence of biological or physiological dysfunction, symptoms and functional impairment (American Thoracic Society 2007:6). It could also be described as the affected individual’s subjective ratings of health perceptions and health status (American Thoracic Society 2007:6).

**Nursing**: A profession within the healthcare sector, focused on the care of individuals, families and communities to attain, maintain or recover optimal health and quality of life (International Council of Nurses 2019:1). Other key roles in nursing are advocacy, promotion of a safe environment, research, participation in shaping health policy and care for patients, health system management and education (International Council of Nurses 2019:1).

**Health**: A state of complete physical, mental and social well-being and not merely the absence of illness or infirmity (Weller 2009:182). In this study, this concept implied the occupational suffering and health status of newly qualified professional nurses. Their development and non-development, including evidenced-based practice in the working area, were observed under this concept, which also measured the participants’ morale against their experiences in the workplace.

The subconcepts described hereunder were used to measure the newly qualified professional nurses’ health needs.

**Human beings:** In this study, this concept refers to all professional nurses who had 2 years of experience as professional nurses, excluding the community service period, specifically the professional nurses who work in hospital facilities in the catchment area that was selected for the study (Webster 2020:1).

In this study, the nursing concept was used as newly qualified professional nurses’ core business at the centre of all other concepts.

The core business is an idealised construct intended to express the organisation’s main or most essential task (Collins English Dictionary 2019).

### Study population

The research population for this study included all newly qualified professional nurses working in hospital facilities in the Alfred Nzo District Municipality.

### Sampling

The study employed purposive sampling (De Vos et al. 2012:228) and 19 participants were interviewed before data saturation was achieved.

### Data collection

The researcher contacted the hospital managers for data collection immediately after the Department of Health and the Walter Sisulu University Research Committee had approved the study. The researcher used interviews, observations and audio material as tools to obtain information from the participants.

### Data analysis

Data analysis was performed according to Tesch’s thematic analysis (1990, as cited in Creswell 2014:186) as described hereunder:

- The data were organised and prepared for analysis, which involved transcribing the interviews, optically scanning the material, typing field notes, and sorting and arranging the data into different types depending on the source of the information. The interviews, containing descriptions of the participants’ experiences, were transcribed one by one.

## Research findings

The research findings revealed that the participants felt bullied in the workplace.

### Theme 1: The participants felt bullied in the workplace

#### Subtheme 1.1: The participants sometimes felt bullied by their seniors

The research participants sometimes felt bullied by their colleagues because they were new to the employment environment. They also felt exploited because of their age because a senior staff member would sometimes remain idle while the newcomers performed all the routine procedures in the unit. Although they were loath to question their colleagues about why they were not assisted with the routine work, they felt that if the ward matrons made rounds unexpectedly to check if all the nurses were tending to patient care, it could reduce bullying in the clinical units. Lindfors and Junttila (2014:3) opine that the experience of incivility and bullying is often associated with the higher job and career turnover intentions, especially among nurses in their first year of practice. The quotes presented hereunder depict how the participants felt about being bullied by their seniors:

‘And another thing that have happened is that when you are new, I sometimes felt bullied because if you are new professional nurse, then the elderly professional nurse will just seat by the nursing station the whole day, in order for you to [*do*] this, to [*do*] that. To do that you will do rounds, you will do injections then you do the books and everything. You do admin duties but she is there also but she doesn’t assist you and we just don’t know why. And it is difficult to even ask sister why am I doing everything alone here? Mmh! Am I the only sister? It’s difficult to ask that because they will say yhoo you are cheeky and all that everything.’ (P6, 22 year old female)

Some of the participants perceived being undermined in front of junior categories of nurses because they were newly qualified professional nurses. This treatment resulted in the juniors refusing some of the work that was delegated to them because they thought there were sufficient suitable staff members on duty to execute those tasks. Ruiters (2020:54) concedes that the bullying behaviour exhibited by the managers belittled the novice nurses. This belittling behaviour included managers speaking rudely to staff members and gossiping about some staff members with the most junior personnel:

‘And I was like why she would delegate me a chamber if she can’t do it herself as a sister too after all? Like why she is not delegating the lower categories, why is she delegating to me?’ (P18, 38 year old female)

The foregoing quotes revealed the bullying behaviour the participants perceived from their senior colleagues. The ensuing discussion focuses on the high expectations of the participants’ colleagues.

#### Subtheme 1.2: The participants felt that their colleagues expected them to know everything despite the minimal experience they had as novice professionals in the service

The participants experienced that they were expected to be experts in the wards because of their 4-year training as nurses. They reported that their colleagues would create an environment where they felt they could not ask questions about how to perform their duties. Several of the participants became frustrated to the extent that they thought they were not worthy to be nurses. Liang, Lin and Wu (2018:74) agree that newly graduated professional nurses are in transition between an environment in which supervision is the norm and somebody would take responsibility for their actions and an environment in which they are expected to make important decisions about nursing matters. They also posit that a reduction in the supervision of the newly graduated nurses increased self-confidence and the courage to accept new tasks in the clinical area. The quotes presented hereunder reveal how the participants felt about being expected to know everything:

‘Eh! Not really, people have been quite good just the fact that sometimes someone would come in, a patient would come in and we kind of we haven’t learnt it in school or we never done or touched on it practical you know. And people just assume that because you are PN [*Professional Nurse*] you must just know what to do you know?’ (P19, 27 year old female)

‘And then sometimes they kind of make you feel you can’t ask because you studied, you are PN now. You should know [*laughing*] but for me, I learnt a lot I learnt a lot with experience on practical so when you studied the textbook is not always like. You see patient that might have one specific thing and then another thing and then you just confused like that’s not what the book says [*laughing*]. So eh yeah!’ (P19, 27 year old female)

‘So you find that, well, you are expected to know everything in the ward, like maybe you supposed to know from A-Z, you supposed to mother everything because they have got this say that those of four year course or those with four year course and everything. Like they want us to be perfect in everything, meanwhile we are like not even supervised in some other things.’ (P18 38 year old female)

‘For other procedures we are not actually supervised, because they will want to delegate you to do something you don’t even know like where do you start. Like how are you supposed to go on with it in order for it to be productive?’ (P18, 38 year old female)

‘Yes! Because now you are expected to know everything but it’s not easy when you go to this ward in this month and you go to the next one, yes, and people expect you to know although they have stayed there for like ten years, when we just came in just for …’ (P14, 27 year old male)

The latter quotes explain how the participants felt when their colleagues expected them to know everything with the minimal experience they had as novice professionals in the service. This theme with its subthemes focused on a description of the negative attitudes the participants experienced at the hands of colleagues and multidisciplinary team members. The next theme focused on the shortage of staff and resources at work that triggered the participants’ feeling ineffective in the work environment.

### Theme 2: The shortage of staff and resources made the participants feel ineffective in their work environment

The shortage of staff in the clinical facilities overwhelmed the participants to such an extent that they would have to multitask to complete all the routine duties allocated by their superiors in the units. They revealed that they were sometimes left alone to run the ward, perform all the necessary tasks and attend facility meetings in the absence of the professionals who were in their category. They perceived this as a significant challenge, as they were still correlating the theory that they had learnt with the practice in the ward. The participants highlighted that the shortage of staff was not only limited to nursing but also affected other general departments of the hospital facilities and they had to pause their tasks and assist where there were shortages. Haddad, Annamaraju and Toney-Butler (2021:8) support the statement and opine that even in the United States, the nursing profession continues to face shortages of staff because of a lack of potential educators, high turnover and inequitable workforce distribution. Lindfors and Junttila (2014:2) admit that while the healthcare sector is struggling with a nursing shortage, nurses are leaving the profession, especially newly graduated nurses. The quotes presented hereunder attest to the shortage of staff:

‘First of all, we are few in this hospital as a professional nurse yet the duties are over us but we manage. Sometimes you become alone in a facility whereby you have to do rounds, doctor’s rounds. You have to give oral medication plus injection and still after that do assessment and also you are expected as a professional nurse to attend daily meetings of the institution. So it’s really a workload that is, that is not good to us.’ (P2, 25 year old female)

‘But then the first challenge I’ve faced is that there is a shortage of staff in this institution. So when I was working as a com serv [*Community Service Nurse Practitioner*] sometimes you were supposed to be left alone and then you had to deal with eeh ward. Eeh you had to correlate the theory that you have learnt at school and then correlate with ee the practical.’ (P9, 26 year old male)

‘So when I came here I would face a patient who has such a problem and I was expected to manage that on my own, without anyone supervising me because they’re also busy. Because of big shortage of staff, there is no one who will be going around with you, showing how things are done. Mmh! So I would be using YouTube to see how it is managed.’ (P11, 45 year old female)

‘Eeh yeah I’ gonna start with first question neh! Mmh! I execute them well besides the fact that there are challenges at work, like for instance, for example neh! At the moment I’m working at OPD [*Out-Patient Department*], the infrastructure there is not good yes. The patients there, we have to take the chairs out so that they can seat there out. And then there is nobody who is there to queue marshal the patients.’ (P15, 41 year old female)

The participants also reported that the inadequate resources in the hospital facilities to execute daily duties were unresolved challenges that forced them to take equipment from other units. The participants expressed their concern about the provision of insufficient care to their patients, which worried them in their holistic healthcare approach. They reported that they sometimes felt helpless when they were unable to satisfy a patient’s health needs. Rivaz et al. (2017:30) posit that inadequate equipment is one of the most vital stumbling blocks in the healthcare setting and leads to disruption, missed or delayed delivery of care and emotional tension. The quotes presented hereunder attest to the shortage of resources:

‘No! We actually use the blood pressure machines but you have to go and fetch it from another ward or borrow it from another ward so that you can come and use it to another ward. Coming to a point where you don’t have glucometer totally in the ward. So the ward has no glucometer. So actually you give minimal treatment to the patient, not the full range of the treatment that you are supposed to give or end up using like certain, depending on doctors to approve taking bloods so that it can be taken to the lab for investigation. So that’s the other thing that is draining about it.’ (P8, 26 year old male)

‘We are emotionally drained, we are physically exhausted. We really need counselling, I mean psychological counselling. We have seen things happening in our wards. We have seen shortage of staff, shortage of equipment, shortage of oxygen and there is nothing you can do while patients are dying stating that Sister umoya uyaphela [*sister I cannot breathe*] [*holding chest with right hand*] and yet there is nothing you can do, there is no oxygen in the unit. There is no oxygen in the facility.’ (P2, 25 year old female)

The shortage of staff and resources in the hospital facilities to which the participants were sent highlighted a significant gap in their support when executing their daily duties. As a result of the shortages described in Theme 2, the following subthemes explained significant moments when the participants felt that they were on their own:

- The participants felt abandoned when they were put in charge of the coronavirus disease 2019 (COVID-19) units alone.
- The participants were afraid and frustrated to be left alone in the units but this created an opportunity to gain clinical experience.

#### Subtheme 2.1: The participants felt abandoned when they were put in charge of the coronavirus disease 2019 units alone

The COVID-19 pandemic was another factor that led to psychological trauma and anxiety for the participants while they were executing their duties in the hospital facilities. They reported that they were left alone to nurse critically ill patients in the COVID-19 units. They reported that several patients died on their watch and for some of the participants, this was their first experience of a patient dying. The participants felt that they were sent to work in COVID-19 isolation units because they were young and new to the profession. They expressed that they felt like they had no say, as their older colleagues refused to work in the isolation units. The latest research proves that older people are at high risk of COVID-19 mortality because of the comorbidities associated with old age (Ho & Petermann-Rocha 2020:1). They expressed the need for professional counselling following the peaked waves that engulfed the Alfred Nzo District Municipality when they were working as novice professionals. The quotes presented hereunder are evidence of the forgoing discussion:

‘Ah! You are … ok there was a case where I was left alone, it was isolation ward during this COVID-19 and it was very hectic because I was alone and the other nurse I was supposed to be with had a personal problem. She had to take a leave. There were many patients in the ward, you have to wear your PPE [*personal protective equipment*] when you get in the ward, you’ll have to resuscitate patients alone because there are very few doctors. It was over the weekend.’ (P9, 26 year old male)

‘Well I have learnt to be strong on my own because there is no one. There is shortage I’m also a witness that there is shortage. So I just have to be strong, waking up going to the unit every day not knowing that I am the only professional nurse, as I’m young as I am. Then I have to face such things. You just have to be strong for patients, not for anyone else but for the patients. Because you are working alone on your own.’ (P2, 25 year old female)

‘Because even during this COVID thing, following COVID isolation. They would get there, the new ones like the new PNs and new like in the staff. The old ones didn’t like to go to those isolation wards … Yes!’ (P18, 38 year old female)

The foregoing quotes focused on the participants’ experiences when they felt abandoned in the COVID-19 units and had to take charge. The ensuing theme explains that the participants were afraid and frustrated to be left alone in the units but that it created an opportunity to gain clinical experience.

#### Subtheme 2.2: The participants were afraid and frustrated to be left alone in the units but it created an opportunity to gain clinical experience

The participants revealed their mixed feelings about working alone in the clinical units. They were afraid to be left alone to perform their duties but at the same time, they realised that it was an opportunity to use their knowledge to manage the patients. They felt a sense of motivation to perform their duties, as some of the people they nursed recognised them as healthcare providers even when they were off duty. The quote presented hereunder attests to this:

‘Eehm, at first it was an excitement because it was like whatever thing we were taught at school, it’s time to put it into practice but in nursing we deal with human lives. So it’s scary because you know whatever that happens, you are going to be accountable for that and you even pray because I remember the patient was going to Mtata. I was praying the whole day that she reach Mtata still alive and I was checking-up until the patient came back. But it was nice because the results were positive. I don’t know if the results would be negative how would I feel but it is emotionally exhausting and we are always praying Jees!’ (P7, 27 year old female)

‘So when I was working as a com serv [*community service*] sometimes you were supposed to be left alone and then you had to deal with eeh ward. Eeh, you had to correlate the theory that you have learnt at school and then correlate with ee the practical. So it was a fortunate thing for us because when you are left alone you are able to manage everything. You are able to delegate others.’ (P9, 26 year old male)

The foregoing quote highlighted the threats and opportunities experienced by the participants when they were left alone in the clinical units without adequate support. The second theme with its subthemes explained the effects of staff and resource shortages in the workplace. The ensuing theme discusses the impact of the participants’ clinical exposure to different units and procedures. It also explains how the exposure added value to the participants’ knowledge and development.

### Theme 3: Clinical exposure to different units and procedures added value to the participants’ development

The participants reported numerous challenges while executing their duties in the hospital facilities in which they were placed that were discussed extensively under Theme 1. The participants reported that the challenges they encountered while performing their duties were often beneficial, as they had added value to their knowledge in the clinical field.

The participants reported that if the challenges mentioned under this theme were adjusted in their work environment, particularly in subtheme 3.1, they would be motivated to perform their duties. They acknowledged the training they received as novice professionals at the hospital facilities and reported a sense of morale when they had to discuss hospital cases with professionals.

A study conducted by Atakro et al. (2019:7) found that neophyte nurses were allowed to come into contact with complex diseases and medical devices as a result of placement in various clinical units in a variety of hospitals. They emphasised that the early exposure of novice professionals to a variety of clinical cases assists those nurses to develop a positive attitude towards patient care, as long as the necessary clinical support system is in place. The quotes presented hereunder illustrate the areas where the participants reported elements of development while performing their duties in the hospital facilities in the Alfred Nzo District Municipality:

‘I’ll start with the good ones that I’ve been placed in the wards that I was never been placed before. So I got the experience there I never had before. I saw different conditions that I never experienced even in my training, so it was good.’ (P5, 27 year old male)

‘Yeah the good experience that I had here, is that eh, we’re usually exposed in many things due to shortage of doctors of which makes us more competent in other things like as you can argue someone theoretically and clinically.’ (P16, 24 year old female)

‘And can hear you and you can be respected with your argument because you know your story. Yeah what I like and a good monitoring. For example, in one ward, in casualty, there is a lot of work there. There’s a ortho [*orthopaedic*], trauma nurses, they are very trained, they are NIMART [*nurse-initiation and management of antiretroviral treatment*] trained nurses. Ok. They usually teach you eh because …’ (P16, 24 year old female)

The aforementioned benefits resulted in a motivating work environment and boosted the participants’ morale while correlating their knowledge with the duties they were executing in the hospital facilities. The following subthemes were explained by the participants:

- Inadequate exposure to clinical learning during training, especially in speciality units, became a challenge when the participants were registered.
- The participants who did not understand some of the units because of a lack of exposure during training became positive about the units during their employment period.
- The participants felt that the difference in the behaviour of senior colleagues in some of the units helped them to understand that not all the professionals were the same; several senior colleagues assisted them with their duties, which motivated them.

#### Subtheme 3.1: Inadequate exposure to clinical learning during training, especially in speciality units, became a challenge when the participants were registered

The participants reported that several problems they experienced during the execution of their duties resulted from their inadequate training. They indicated that if some of the problems, such as clinical placement, were resolved during training, the experiences discussed under this subtheme would have been more positive. The participants claimed that the training institutions focused on theory more than practice, which resulted in a gap in their capacity when they qualified as registered nurses.

Jamshidi et al. (2016:240) also emphasise inadequate exposure to clinical learning and stress that optimal clinical learning has a positive impact on the learners’ professional development and that a poor learning environment can have adverse effects on their professional development process. The participants spoke about their experiences concerning clinical exposure in their training and the clinical units as novice professionals:

‘No it’s not. It’s not ok. It’s not because here we’ve got the time to rotate around and then get to know like other wards. Though we didn’t know them deeper but ok this what it’s happening in this ward, because we rotating once only a month. But for me it became challenge when I was placed in high care and I was placed in theatre yet as a student I was never been to theatre. So it became like that.’ (P6, 22 year old female)

‘It was occurring in clinical and also in theory, yeah in class. Because even in class it’s about marks, it’s about you should get distinction. It’s not a bad thing that we should get good marks but I feel like we should focus more on practical than on theory. Because there are students who are very good in practical but who are still in the course because they don’t performing well in theory. So I don’t know how to make it or practical be more prominent than theory because at school it was about marks.’ (P7, 27 year old female)

‘Mmmhh the other challenge I faced it was eeehmm, ok the other thing is that as we rotate as professional, as comm serv [*community service*] professional nurses we don’t get a chance to go to other wards. Sometimes the change list would come and tell you’re repeating the very same ward. For instance I have never been exposed to paeds [*paediatric unit*]. Unfortunately even when I was a student I was never exposed to paeds so I’m very scared because change list this year is changing like suddenly, every month you go to other ward, so like I’m not feeling ok because I’m gonna be like a professional nurse who knows nothing.’ (P9, 26 year old male)

The foregoing subtheme explains how inadequate exposure to clinical learning, especially in speciality units during the training, became a challenge when the participants were registered. The ensuing description refers to the mindset shift from the students’ negativity period to the positivity period following exposure to practical work after being employed.

#### Subtheme 3.2: The participants who did not understand some of the units because of a lack of exposure during training became positive about the units during their employment

The participants’ attitudes and behaviour changed positively when they were placed in the clinical units for extended periods, compared with their initial placement when they were not well-oriented in the units. This extended placement allowed them to gain a sense of responsibility in the execution of their duties. Some of the participants were even keen to specialise in some of the units that they disliked before their community service commenced. Geue (2018:300) holds that positivity in an organisation yields enhanced results and leads to positive behaviour among the employees and enhanced organisational performance. The quotes presented hereunder reveal the participants’ attitudes when they were first placed in the clinical units and after they had adapted to these units’ routines:

‘Yes a huge, a huge difference, like it’s a huge difference now, even my lecturers if they can come here, I’m sure they will think that they will get the bad record of me not coming to work but [*laughing*] I was not this good at school but they will meet the opposite results because here it’s not about … nobody forces you to do anything, you see the need of doing it. Even now that I’m here, I know that my ward because I’m allocated in postnatal, everything is waiting for me to go back there.’ (P7, 27 year old female)

‘So I know when I’m from here I have to go back to the ward but if I was at school I would take this chance as a chance of dodging and going to town. And then when they asking me where were you? I would say aah I was just doing the interview with Mr. mmh! So there’s a huge difference, the reason for that it’s because the environment here is relaxed and people they trust you. They believe you to do whatever, so at school they always have that …’ (P7, 27 year old female)

‘Mmmh yeah! Eeh actually I would say it was good experiences because I never like … for instance, I ended up developing love with kids, with small babies because aah … Am I allowed to mention units? Yeah! Yes like on my comm serv [*Community Service Nurse Practitioner*] I was working in paediatric ward. I was very scared of baby who is sick but I developed some love and eeh you know some understanding you see. And when I was being absorbed, I then moved to maternity. So maternity I worked mostly in labour ward, so even midwifery is … it was a … I didn’t like it I was just doing it because it was part of programme you see but I didn’t like it. But for this I think eeeh it’s approaching the year now because I started in April last year in maternity now its march so it’s almost a year. So I find that very, very, you know, educative and interesting so yah, I’ll say it’s good, it’s good because I developed love for working with small babies and love for midwifery because midwifery even as a module I didn’t like but now I can even advance, you know I can even advance.’ (P4, 31 year old male)

‘But for me I learnt a lot, I learnt a lot with experience on practical so when you studied the textbook is not always like. You see patient that might have one specific thing and then another thing and then you just confused like that’s not what the book says [*laughing*]. So eh yeah!’ (P19, 27 year old female)

The foregoing subtheme explained that the participants did not understand some of the units during their training but changed their attitudes positively during their employment period. The ensuing subtheme focuses on the participants’ analysis of the work environment and the assistance they received from their colleagues, which motivated them.

#### Subtheme 3.3: The participants felt that the difference in the behaviour of senior colleagues helped them understand that not all professionals are the same; several senior colleagues assisted them with their duties, which motivated them

Although the participants sensed their senior colleagues’ negative attitudes, they also observed that some staff members were always willing to assist them as novice professionals in nursing. The participants would seek assistance from these senior professional nurses, who had a supporting and welcoming attitude towards them until they grasped how things were performed in that particular clinical unit. Milliard (2020:389) reiterates that staff members perceive peer support as more than just a conversation but also a method of increasing their intellectual literacy. The quotes presented hereunder portray how the newly qualified professional nurses perceived the assistance they received from some staff members in the clinical units:

‘So you find, ok this person and this person with attitude so you always go to this one and will say its fine, let me help you, because I know you are new here in the experience.’ (P1, 27 year old male)

‘It was great, it was great eeh I felt like I was not alone. They understand that I’m new yes, I am a new nurse yes that was good. Mmh! So as there were those experiences but I had assistance, yes and they had a level of understanding yes.’ (P14, 27 year old male).

‘We do allocation in the morning and people stick to it and people do things like if I’m busy with something else but I meant to do something they just follow up and do it.’ (P19, 27 year old female).

The foregoing quotes illustrated the participants’ analysis of their work relationships and the development that was facilitated by some of their colleagues. Theme 3 elaborated on the participants’ experiences following their exposure to various units, particularly speciality units in which they felt that they had limited exposure before they qualified as practitioners. In this last theme, the participants explained their development through adequate exposure to clinical work.

## Recommendations

- Hospital manager should place policy measures to guard against bullying of newly qualified nurses in the workplace.
- Hospital management should employ more staff who would help in orientation of new staff.
- Resources should be made available to assist health workers in the delivery of healthcare.
- Mentors and coaches should be allocated to each newly employed nurse.

## Conclusion

The research revealed that newly qualified professional nurses experienced disrespect from their colleagues when they delegated duties to both senior and subordinate staff members. This attitude led to the newly qualified professional nurses’ feelings of anger and resentment. The disrespect they experienced was accompanied by insubordination. This behaviour increased the stress from overworking and tending to their responsibilities and duties.
